# Serum Bovine Immunoglobulins Improve Inflammation and Gut Barrier Function in Persons with HIV and Enteropathy on Suppressive ART

**DOI:** 10.20411/pai.v4i1.276

**Published:** 2019-05-03

**Authors:** Netanya S. Utay, Anoma Somasunderam, John E. Hinkle, Bryon W. Petschow, Christopher J. Detzel, Ma Somsouk, Carl J. Fichtenbaum, Eric M. Weaver, Audrey L. Shaw, David M. Asmuth

**Affiliations:** 1 Department of Medicine, McGovern Medical School, University of Texas Health Science Center at Houston, Houston, Texas; 2 EarlyPhase Sciences, Inc., Magnolia, Texas; 3 Entera Health, Inc., currently located at 2425 Oak Tree Ct., Ankeny, Iowa; 4 Department of Medicine, University of California, San Francisco, San Francisco, California; 5 Department of Medicine, University of Cincinnati, Cincinnati, Ohio; 6 Department of Medicine, University of California Davis Medical Center, Sacramento, California

**Keywords:** HIV infection, CD4 T cell, Serum bovine immunoglobulin protein, Interleukin, Inflammation, Intestine, I-FABP

## Abstract

**Background:**

Systemic inflammation persists in chronic HIV infection and is associated with increased rates of non-AIDS events such as cardiovascular and liver disease. Increased gut permeability and systemic exposure to microbial products are key drivers of this inflammation. Serum-derived bovine immunoglobulin/protein isolate (SBI) supports gut healing in other conditions such as inflammatory bowel disease.

**Methods:**

In this randomized, double-blind study, participants receiving suppressive antiretroviral therapy (ART) with chronic diarrhea received placebo or SBI at 2.5 g BID or 5 g BID for 4 weeks, followed by a 20-week placebo-free extension phase with SBI at either 2.5 or 5 g BID. Intestinal fatty acid binding protein (I-FABP), zonulin, flagellin, lipopolysaccharide (LPS) and LPS-binding protein, and inflammatory markers were measured by ELISA or multiplex assays. Non-parametric tests were used for analysis.

**Results:**

One hundred three participants completed the study. By week 24 SBI significantly decreased circulating levels of I-FABP (-0.35 ng/μL, *P*=0.002) and zonulin (-4.90 ng/μL, *P=*0.003), suggesting improvement in gut damage, and interleukin-6 (IL-6) (-0.40 pg/μL, *P*=0.002), reflecting improvement in systemic inflammation. In participants with the lowest quartile of CD4+ T-cell counts at baseline (189-418 cells/μL), CD4+ T-cell counts increased significantly (26 cells/μL; *P*=0.002).

**Conclusions:**

Oral SBI may decrease inflammation and warrants further exploration as a potential strategy to improve gut integrity and decrease systemic inflammation among persons receiving prolonged suppressive ART.

## INTRODUCTION

Human immunodeficiency virus (HIV) infection is characterized by profound depletion of CD4+ T cells systemically and in the gastrointestinal (GI) tract, compromised mucosal barrier function, translocation of microbial products, and chronic inflammation [1]. Suppressive antiretroviral therapy (ART) decreases but does not normalize inflammatory biomarkers [2, 3]. Consequently, diseases associated with increased inflammation are more common in people with HIV, including cardiovascular events, non-AIDS malignancies, liver disease, and others [4].

A key contributor to this chronic inflammation is increased translocation of microbial products across a permeable gut barrier from the intestinal lumen to the lamina propria and systemic circulation [1, 2]. These microbial products activate innate immune cells to produce pro-inflammatory cytokines, such as interleukin-1β (IL-1β), interleukin-6 (IL-6), and tumor necrosis factor-a (TNF-α) [1]. Indeed, circulating markers of intestinal permeability (intestinal fatty acid binding protein [I-FABP] and zonulin) and IL-6 have been consistently predictive of non-AIDS events and mortality in people receiving suppressive ART [2, 5]. Thus, an intervention that decreases intestinal permeability and systemic inflammation, and its drivers, could decrease the excess morbidity and mortality associated with well-treated HIV infection.

Gut inflammatory markers are decreased in animal models of colitis after oral administration of plasma protein preparations with high levels of immunoglobulins [6, 7]. Serum-derived bovine immunoglobulin/protein isolate (SBI) is an oral powder containing high concentrations of immunoglobulin that is not absorbed. It is the primary ingredient in a medical food that has been used successfully to manage patients with diarrhea-predominant irritable bowel syndrome (IBS-D) and inflammatory bowel disease [8-10]. SBI is enriched for immunoglobulins, comprised of >50% IgG, 1% IgA, and 5% IgM, which bind lipopolysaccharide (LPS) or endotoxin and other conserved microbial antigens [11-13], in addition to amino acids, albumin, and transferrin[14]. In a previous study involving 8 patients with HIV-associated diarrhea, we found improvements in GI symptoms, lymphocyte populations in gut-associated lymphoid tissue (GALT), and markers of intestinal repair following 8 weeks of SBI therapy [15]. In a follow up study, we found improvements in stool frequency and several other GI symptoms but with a greater than expected placebo effect [16]. Here, we aim to report the impact of oral SBI on biomarkers of intestinal integrity and systemic inflammation in virologically suppressed persons with HIV with gastrointestinal symptoms.

## METHODS

### Study Population

Adult men or non-pregnant women with HIV and virologic suppression for at least 12 months were eligible if they had enteropathy; this was defined as 3 or more loose stools per day for at least 3 months. Participants were not eligible to participate if they had (1) a positive stool test for pathogenic bacteria, ova, or parasites during the 14-day screening period, (2) changes in antiretroviral medications during the 3-month period prior to screening, or (3) a condition that required chronic therapy that might alter the gut flora or the use of an antibiotic within 2 weeks prior to screening. Institutional Review Boards at each site reviewed and approved the protocol [Clinical Trial Registry Number Identifier (Clinicaltrials.gov): NCT01828593], and all participants provided written informed consent.

### Design

**Supplementary Figure 1.**
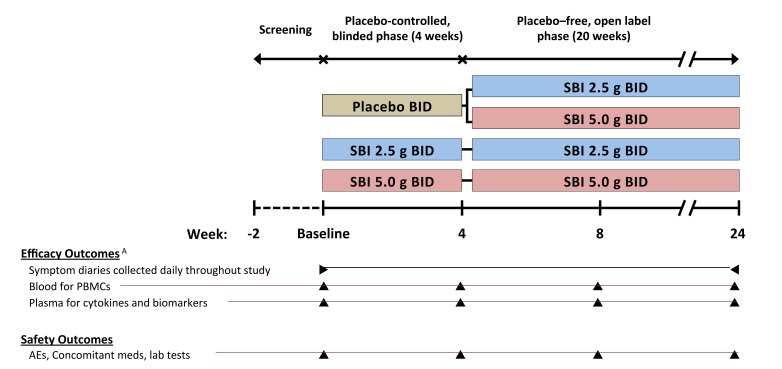
Study design and schedule of outcome procedures.

This prospective, multicenter, randomized, blinded study included a partial cross-over design comprising 2 study phases: a double-blind placebo (PBO)-controlled phase and a placebo-free blinded extension phase. Participants were randomized at study entry to (1) placebo BID for 4 weeks followed by either twice daily LD-SBI (low dose-SBI, 2.5 g) for 20 weeks (n=21) or HDSBI (High dose-SBI, 5 g) for 20 weeks (n=14); (2) Twice daily LD-SBI for 24 weeks (n=34); or (3) Twice daily HD-SBI for 24 weeks (n=33; Supplementary Figure 1). Participants received placebo (2.5 g dextrose) or SBI dissolved in 120 μL of water and remained blinded to their treatment assignment–whether they had received the placebo lead-in or low dose versus high dose assignment for the duration of the study. Thirteen of 103 randomized participants did not complete the study as previously described [16]. All study participants had blood samples obtained at baseline and weeks 4, 8, and 24 to evaluate PBMCs and biomarkers of bacterial translocation, inflammation, enterocyte damage, coagulation, and pro-inflammatory cytokines.

### Parent Clinical Trial Outcomes

The primary efficacy endpoint of the parent clinical trial was the change in number of bowel movements (BM) per day from baseline to week 4, as previously reported [16]. Predetermined exploratory endpoints included changes in peripheral CD4+ and CD8+ T-cell counts, plasma markers of intestinal damage, microbial translocation, inflammation, and coagulation at week 4 and last observation. Peripheral CD4+ and CD8+ T-cell counts were measured by flow cytometry using standardized methods (North Coast Clinical Laboratory, Inc., Sandusky, OH).

### Measurement of Cytokines and Biomarkers for Microbial Rranslocation and Intestinal Permeability

The level of LPS was assayed in duplicate using a Lonza LAL QCL-1000 kit (Lonza, Walkersville, MD) according to the manufacturer's protocol. LPS, LPS-binding protein (LBP), and Bactericidal/Permeability Increasing Protein (BPI) were measured in plasma samples using enzyme-linked immunosorbent assay (ELISA)-based assays according to the manufacturer's protocol (MyBio-Source, Inc., San Diego, CA), and flagellin was measured by ELISA in serum (USCN Life Science Inc., Missouri City, TX).

Levels of pro-inflammatory cytokines (IL-1β, TNF-α), Th1 (interferon-γ [IFN-γ] and IL-12_p70_), Th2 (IL-4), and regulatory cytokines (IL-10), chemokines (IL-8), and monocyte chemoattractant protein-1 (MCP-1 or CCL2), and biomarkers of collagen regulation (hyaluronic acid (HA), transforming growth factor-β (TGF-β) 1, 2, and 3, matrix metalloproteinases (MMP)-1, MMP-9, tissue inhibitor of metalloproteinases (TIMP-1, and TIMP-2) were all measured in plasma with multiplex ELISA-based assays (MesoScale Discovery; assay kit for HA from Corgenix, Broomfield, CO). Serum levels of IL-6 and soluble CD14 (sCD14) were measured with Magnetic Luminex^®^ Kits (R&D Systems, Minneapolis, MN).

Serum intestinal fatty acid binding protein (I-FABP) is an intestinal isoform of a ubiquitous intra-cellular protein that is known to be elevated in diseases of chronic intestinal inflammation such as celiac disease as well as in people with HIV and chronic diarrhea. I-FABP was measured using an ELISA–based assay (R&D Systems, Minneapolis, MN). Serum zonulin levels were measured using a competitive ELISA method (Immundiagnostik AG, Bensheim, Germany).

### Statistical Methods

The analysis used change from baseline measures to the end of week 4 of the PBO-controlled phase of the study and change from baseline to last observation carried forward (LOCF) in the 20-week PBO-free phase of the study from all available double-blind data without imputation of missing data. LOCF data encompasses participants who received SBI for 24 weeks (LD-SBI and HD-SBI) or 20 weeks (PBO crossover at Week 4). A 2-sided significance test using α of 0.05 was used to declare statistical significance and flag results for further inquiry. Changes from baseline data were analyzed using Wilcoxon signed-rank tests of the paired samples. As these analyses were exploratory, no corrections for multiplicity were implemented, and all significant results are considered hypothesis generating [17]. All statistical analyses were performed with SAS software version 9.3 (SAS Institute Inc, Cary, NC).

## RESULTS

### Study Population

Characteristics of the participants at study entry have been described previously and are summarized in Table 1 [16]. The median age was 50 years, with 69% male, 61% African-American and 37% White participants. Median time since HIV diagnosis was 18.2 years, with a median duration of ART of 8.33 years and duration of HIV-associated diarrhea of 3.5 years. Overall, median HIV-1 RNA level was undetectable (defined as 19 copies/μL) across all arms, and CD4+ T-cell count was 637 cells/mm^3^, with no difference between arms (PBO–median 523 cells/μL [Q1 397, Q3 921], LD-SBI–813 cells/μL [469, 939] and HD-SBI–672 cells/μL [478, 776]). The distribution at baseline in the top 3 CD4+ T-cell quartiles (> 418 cells/μL) was near or in the normal range for people taking chronic suppressive ART.

**Table 1.** Baseline Characteristics for Randomized Treatment Groups

		n (%) or median (Q1-Q3)			
Characteristic		Placebo	SBI 2.5 g	SBI 5.0 g	Total
Study Subjects ^a^		36	34	33	103
Sex	Male	28 (78)	21 (62)	22 (67)	71 (69)
	Female	8 (22)	13 (38)	11 (33)	32 (31)
Race	African American	21 (58)	20 (59)	22 (67)	63 (61)
	White	14 (39)	14 (41)	10 (30)	38 (37)
	Asian	1 (3)	0 (0)	1 (3)	2 (2)
Age (years)		50 (34–66)	49 (34–70)	48 (32–65)	50 (32–70)
Time since HIV diagnosis (years)		16.7 (1.8–27.5)	16.4 (6.3–29.5)	19.2 (4.8–28.5)	18.2 (1.8–29.5)
Peripheral CD4+ T-cell count (cells/μL) ^b^		523 (194–1224)	813 (189–1611)	672 (202–1754)	637 (189–1754)
Plasma Viral Load (copies/μL) ^c^		19 (19–64)	19 (19–119)	19 (19–168)	19 (19–168)
Time on ART (years)		7.46 (1.0–23.07)	9.05 (1.0–23.67)	9.89 (1.0–23.73)	8.33 (1.0–23.73)
Time with HIV-associated Diarrhea (years), median (Q1-Q3)		2.2 (0.2–23.7)	4.7 (0.2–29.5)	5.5 (0.1–23.5)	3.5 (0.1–29.5)

a Analysis population = all randomized participants receiving at least 1 dose of investigational product during placebo-controlled phase.

b Quartile ranges were as follows: Q1: ≤418; Q2: > 418 to ≤ 630; Q3: > 630 to ≤ 893; Q4: > 893

c Limit of detection for plasma viral load was 20 copies/μL

Abbreviations: SBI, Serum-derived bovine immunoglobulin/protein isolate; ART, antiretroviral therapy

### Evaluation of Biomarkers of Enterocyte Function, Microbial Translocation, and Inflammation

**Figure 1.**
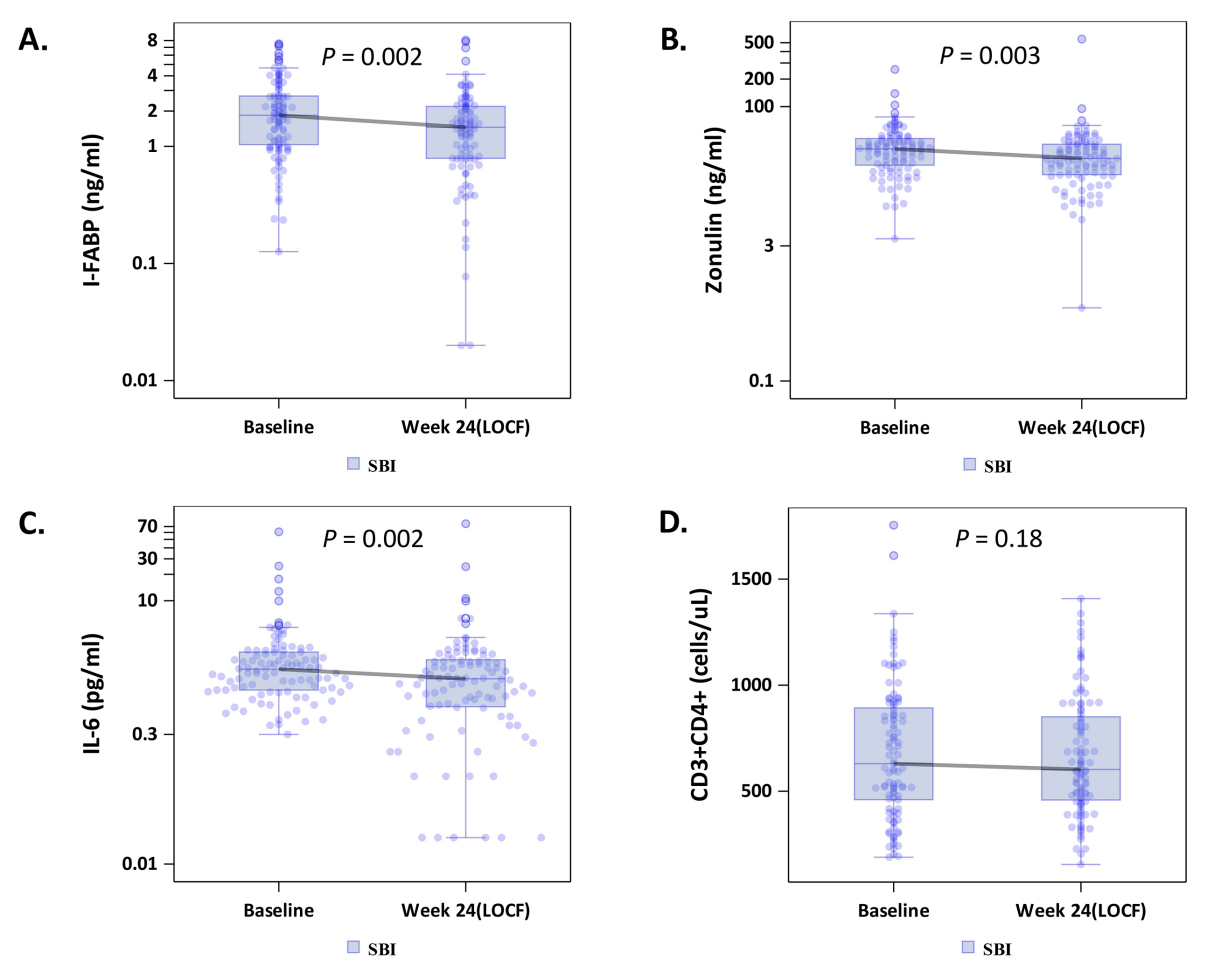
Effects of serum bovine immunoglobulin/protein isolate (SBI) on biomarkers of intestinal permeability (A-B; intestinal fatty acid binding protein [I-FABP], zonulin), (C) inflammation (IL-6) and (D) CD4+ T cell counts among all participants.

**Figure 2.**
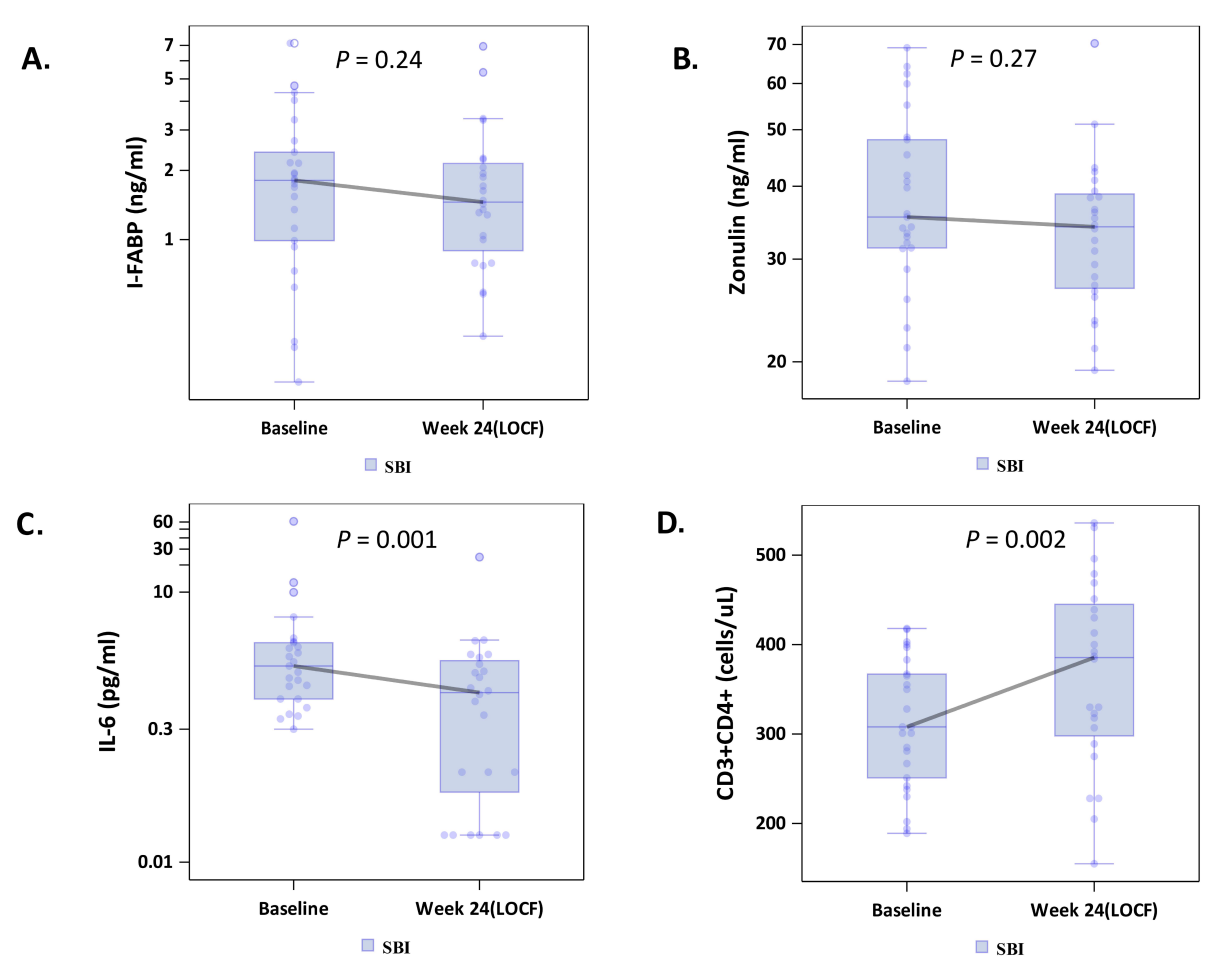
Effects of serum bovine immunoglobulin/protein isolate (SBI) on biomarkers of intestinal permeability (A-B; intestinal fatty acid binding protein [I-FABP], zonulin), (C) inflammation (IL-6) and (D) CD4+ T-cell counts among participants with the lowest quartile of CD4+ T-cell counts.

To explore whether SBI administration would improve gut barrier function, we measured circulating I-FABP levels, reflecting enterocyte turnover [18], and zonulin levels, reflecting tight junction integrity [19]. Median levels of both I-FABP, -0.35 ng/μL (-1.46, +0.31; *P* = 0.002), and zonulin, -4.90 ng/μl (-18.43, +4.51; *P* = 0.003), decreased in the combined SBI groups (LD-SBI and HD-SBI, n=100) (Table 2; Figures 1A and B); changes within treatment groups at week 4 were not significant (Supplementary Table 1). I-FABP and zonulin levels decreased in both SBI dose groups, with a trending dose effect (Table 3). Circulating markers of microbial translocation, however, including LPS, LBP, 16S rDNA, flagellin, and sCD14 (Tables 2 and 3 and not shown) did not change significantly with SBI therapy. As lower CD4+ T-cell counts are associated with more gut damage and microbial translocation [2], we evaluated the lowest baseline CD4+ T-cell quartile (189-418 cells/μL; n=25) separately. I-FABP, zonulin, and flagellin changes decreased without statistical significance in this group, although the small sample size might have limited the power to detect a change (Table 4; Figure 2A-B).

**Table 2.** Within-Group comparison for Combined SBI Groups^a^

Variable	Treatment Group^b^	n Median (Q1-Q3)						*P*-value^d^
		Baseline		LOCF^c^		Delta		
CD4 (cells/uL)	SBI	101	630 (460–893)	100	602.50 (458.50–851)	99	-14 (-119–80)	*0.177*
CD4/CD8	SBI	101	0.71 (0.47–1.17)	100	0.72 (0.49–1.17)	99	0 (-0.07–0.07)	*0.997*
I-FABP (ng/μL)	SBI	101	1.84 (1.03–2.69)	100	1.46 (0.79–2.19)	99	-0.35 (-1.46–0.31)	*0.002*
Zonulin (ng/μL)	SBI	101	34.45 (22.87–44.74)	100	27.09 (18.04–38.79)	99	-4.90 (-18.43–4.51)	*0.003*
Flagellin (ng/μL)	SBI	101	4.76 (2.97–7.23)	100	4.62 (2.52–7.20)	99	-0.18 (-2.66–1.76)	*0.360*
sCD14 (ng/μL)	SBI	101	1.96 (1.62–2.23)	100	1.89 (1.56–2.16)	99	-0.03 (-0.28–0.24)	*0.375*
IL-6 (pg/μL)	SBI	101	1.66 (0.96–2.60)	100	1.29 (0.62–2.13)	99	-0.40 (-1.25–0.35)	*0.002*

a Analysis population = Participants receiving SBI for 24 weeks and participants crossing-over from PBO to SBI after week 4

b Combined SBI treatment groups

c Last observation carried forward, to 24 weeks for SBI-treated group and to 20 weeks for PBO-SBI crossover group

d Wilcoxon Signed-Rank test

Abbreviations: SBI, Serum-derived bovine immunoglobulin/protein isolate; LOCF, Last observed carried forward; I-FABP, intestinal fatty acid binding protein; sCD14, soluble CD14

**Table 3.** Within-Group comparison for SBI Groups^a^

Variable	Treatment Group	n Median (Q1-Q3)						P-value^c^
		Baseline		LOCF^b^		Delta		
CD4 (cells/uL)	SBI 2.5 g	54	803 (469–958)	55	638 (466–915)	54	-38 (-158–67)	*0.018*
	SBI 5.0 g	47	543 (757–417)	45	567 (413–800)	45	16 (-67–83)	*0.539*
CD4/CD8	SBI 2.5 g	54	0.76 (0.46–1.42)	55	0.81 (0.47–1.35)	54	0 (-0.06–0.08)	*0.772*
	SBI 5.0 g	47	0.66 (1–0.48)	45	0.65 (0.51–0.98)	45	0.01 (-0.07–0.06)	*0.760*
I-FABP (ng/μL)	SBI 2.5 g	54	1.95 (0.99–3.11)	55	1.40 (0.79–2.24)	54	-0.23 (-1.48–0.24)	*0.023*
	SBI 5.0 g	47	1.81 (1.10–2.66)	45	1.56 (1–2.10)	45	-0.45 (-1.17–0.36)	*0.043*
Zonulin (ng/μL)	SBI 2.5 g	54	34.61 (19.43–42.24)	55	29.25 (20.59–38.24)	54	-4.16 (-17.07–6.85)	*0.117*
	SBI 5.0 g	47	34.27 (24.16–49.35)	45	26.42 (17.33–40.95)	45	-6.18 (-21.44–3.03)	*0.009*
Flagellin (ng/μL)	SBI 2.5 g	54	4.34 (2.55–7.16)	55	4.22 (1.83–6.61)	54	-0.22 (-2.61–1.55)	*0.307*
	SBI 5.0 g	47	5.14 (3.15–8.02)	45	5.36 (3.42–7.87)	45	-0.07 (-2.66–1.76)	*0.792*
sCD14 (ng/μL)	SBI 2.5 g	54	2.02 (1.70–2.27)	55	1.99 (1.69–2.20)	54	-0.08 (-0.29–0.26)	*0.433*
	SBI 5.0 g	47	1.87 (1.41–2.20)	45	1.79 (1.43–2.05)	45	0 (-0.28–0.21)	*0.675*
IL-6 (pg/μL)	SBI 2.5 g	54	1.72 (1.20–2.71)	55	1.27 (0.65–2.07)	54	-0.53 (-1.27–0.25)	*0.012*
	SBI 5.0 g	47	1.37 (0.91–2.46)	45	1.31 (0.60–2.18)	45	-0.38 (-1.13–0.40)	*0.072*

a Analysis population = Patients receiving SBI for 24 weeks and patients crossing-over from PBO to SBI after week 4

b Last observation carried forward, to 24 weeks for SBI-treated group and to 20 weeks for PBOSBI cross-over group

c Wilcoxon Signed-Rank test

Abbreviations: SBI, Serum-derived bovine immunoglobulin/protein isolate; LOCF, Last observed carried forward; I-FABP, intestinal fatty acid binding protein; sCD14, soluble CD14

**Table 4.** Within-Group Comparison for Combined SBI Groups^a^ in the first CD4+ T-cell Quartile^b^.

Variable	Treatment	n Median (Q1-Q3)						P-value^d^
		Baseline		LOCF^c^		Delta		
CD4 (cells/uL)	SBI	25	308 (251–367)	24	385.50 (298–445)	24	25.50 (-0.50–125)	*0.002*
CD4/CD8	SBI	25	0.38 (0.24–0.53)	24	0.33 (0.27–0.57)	24	0.01 (-0.06–0.06)	*0.848*
I-FABP (ng/μL)	SBI	25	1.81 (0.99–2.40)	24	1.46 (0.90–2.15)	24	-0.35 (-0.78–0.30)	*0.244*
Zonulin (ng/μL)	SBI	25	35.42 (31.35–48.07)	24	34.08 (26.74–38.79)	24	-1.93 (-15.89–4.63)	*0.274*
Flagellin (ng/μL)	SBI	25	5.04 (2.53–7.17)	24	4.78 (3.04–5.86)	24	-0.57 (-2.06–0.94)	*0.122*
sCD14 (ng/μL)	SBI	25	1.78 (1.40–2)	24	1.69 (1.40–1.94)	24	0.01 (-0.30–0.25)	*0.825*
IL-6 (pg/μL)	SBI	25	1.51 (0.65–2.74)	24	0.77 (0.06–1.73)	24	-0.57 (-1.26–-0.16)	*0.001*

a Analysis population = Participants receiving SBI for 24 weeks and participants crossing-over from PBO to SBI after week 4

b Analysis subpopulation = participants in first CD4+T-cell quartile ranges were as follows: Q1: ≤418 (cell/uL) at baseline. Q2-Q4 are: > 418 to ≤ 630, > 630 to ≤ 893, and > 893, respectively

c Last observation carried forward, to 24 weeks for SBI-treated group and to 20 weeks for PBOSBI cross-over group

d Wilcoxon Signed-Rank test.

Abbreviations: SBI, Serum-derived bovine immunoglobulin/protein isolate; LOCF, Last observed carried forward; I-FABP, intestinal fatty acid binding protein; sCD14, soluble CD14

Because IL-6 is a marker of inflammation that correlates with clinical outcomes [5], we measured the impact of SBI therapy on IL-6 levels. The median serum IL-6 level among all participants decreased significantly from baseline to last observation carried forward by -0.40 pg/μL (-1.25, +0.35; *P* = 0.002, n=100) (Table 2; Figure 1C), with no obvious dose effect (Table 3). Notably, 83% of people in the lowest baseline CD4+ quartile had decreases in serum IL-6 levels, with a median change among all participants in this quartile of -0.57 pg/μL (-1.26, -0.10; *P* = 0.001, n=24; Table 4; Figure 2C).

### Peripheral CD4+ T-cell Measurements

Among the combined SBI groups, peripheral CD4+ T-cell counts did not change (Table 2 and Figure 1D). Peripheral CD4+ T-cell counts decreased in participants who received LD-SBI from 803 to 638 cells/μL (-38 cells/μL [-158, +67], *P* = 0.018; n=54), but not in those receiving HD-SBI (+16 cells/μL [-67, +83], *P* = 0.539; n=45) between baseline and last observation carried forward (Table 3). Changes in CD8+ T cells correlated with changes in CD4+ T cells (r=0.74, *P* < 0.0001; data not shown). Consequently, no significant changes were observed in CD4/CD8 T-cell ratios from baseline to week 24 for any of the treatment groups (Table 3).

We examined changes in CD4+ T-cell counts in the lowest quartile of CD4+ T-cell counts because people who have failed to normalize CD4+ T-cell counts may have the greatest potential to experience an immunologic benefit from attenuating systemic inflammation. Among participants in the lowest baseline CD4+ T-cell quartile (189-418 cells/μL; n=25), peripheral CD4+ T-cell counts for the combined SBI groups (LD-SBI+HD-SBI) increased from baseline (308 cells/μL) to last observation carried forward (386 cells/μL) after 20-24 weeks of SBI, with a median change of 25.5 cells/μL (-0.5, +125; *P* = 0.002) (Table 4; Figure 2D); 75% of participants had an increase in CD4+ T-cell counts. This increase was paralleled by an increase in CD8+ T-cell counts of 109 cells/μL (+8, +280; *P* = 0.004; data not shown), from 806 to 964 cells/μL, with 79% of participants having an increase in CD8+ T-cell counts. The CD4/CD8 ratio, however, did not change significantly from baseline (0.38) to last observation (0.33), with a median change of 0.01 (-0.06, +0.06; *P* = 0.85; Table 4). Thus, CD4+ and CD8+ T-cell counts increased significantly during SBI treatment in participants with the lowest CD4+ T-cell counts.

**Figure 3.**
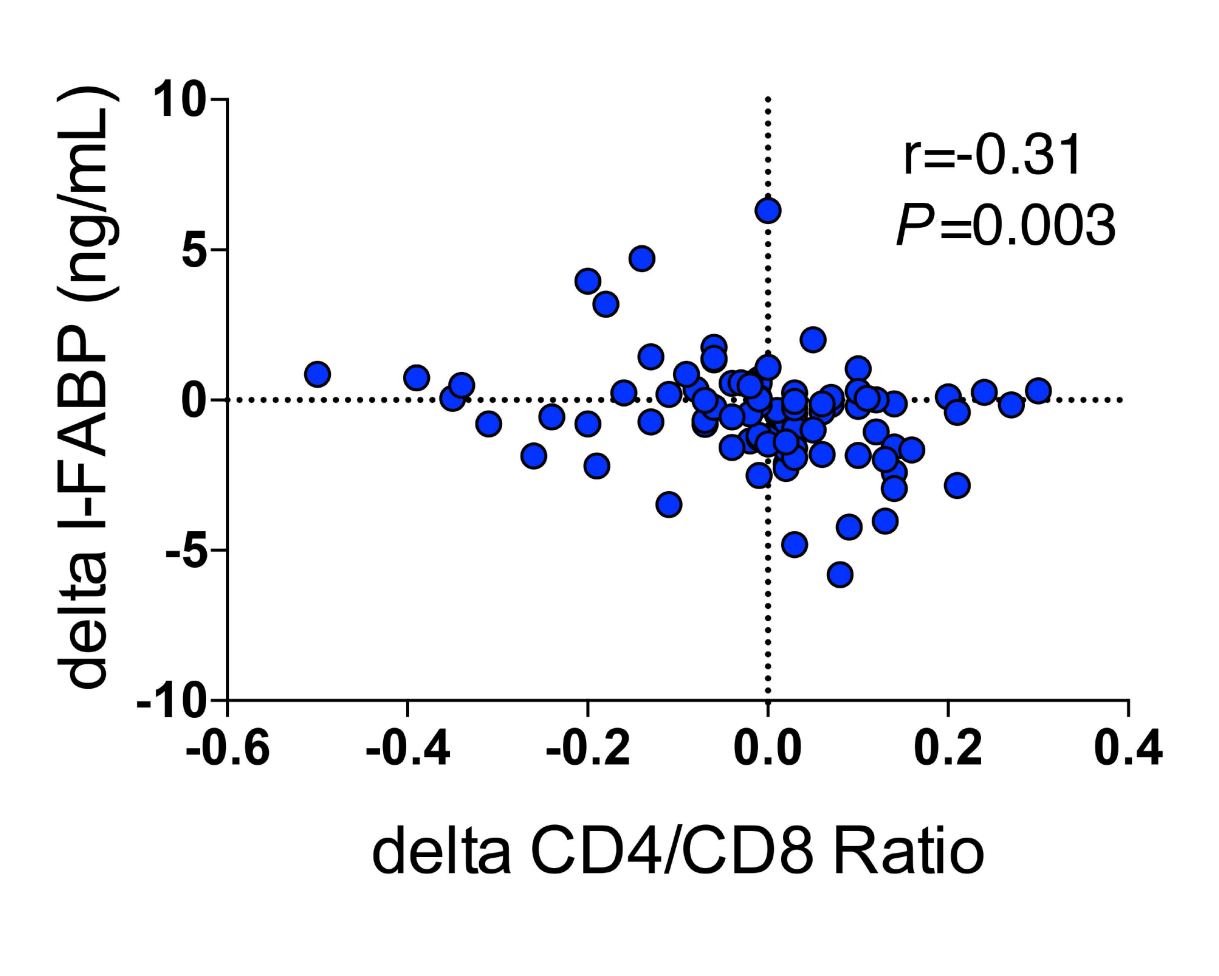
Association of changes in I-FABP with changes in CD4/CD8 T-cell ratios.

Next, we evaluated associations between changes in biomarker levels (I-FABP, zonulin, IL-6, sCD14) and immunologic and clinical parameters from baseline to last observed measurement using Spearman correlations. Changes in flagellin (r= -0.40, *P* < 0.0001), zonulin (r= -0.62, *P* < 0.0001), I-FABP (r= -0.65, *P* < 0.0001), and IL-6 (r= -0.56, *P* < 0.0001) correlated with baseline levels of each respective biomarker, and changes in serum I-FABP levels were associated inversely with changes in CD4/CD8 ratios among all participants (r= -0.307, *P* = 0.003; n=100) (Figure 3). Changes among biomarkers of gut damage did not correlate with changes in biomarkers of inflammation. Participants who had decreases in sCD14 levels tended to be younger (r=0.21, *P* = 0.04) and to have decreases in the frequency of loose stool (*P* = 0.06). We saw no associations between changes in other biomarkers or CD4+ T-cell count and age or frequency of stools. For the lowest CD4+ T-cell quartile group, decreases in IL-6 levels were associated with decreases in sCD14 levels (r=0.535, *P* = 0.008; n=24). In addition, for the lowest CD4+ T-cell quartile group, lower IL-6 levels at week 24 were associated with higher CD4/CD8 ratios (r= -0.516, *P* = 0.012). Thus, I-FABP, zonulin, and IL-6 levels decreased with SBI, and decreases in markers of intestinal permeability and inflammation correlated with increases in CD4/CD8 ratios.

## DISCUSSION

We previously showed that SBI is safe and well-tolerated in people with HIV receiving ART and with chronic diarrhea [16]. People treated with SBI had decreased diarrhea and other GI symptoms but without significant differences from the placebo group. Here, we explored the effects of SBI on biomarkers of gut damage, microbial translocation, and inflammation, and on CD4+ T-cell recovery, as predefined exploratory endpoints. We found that SBI treatment was associated with 1) decreases in I-FABP and zonulin levels, 2) decreases in IL-6 levels, particularly among those with the lowest baseline CD4+ T-cell counts, and 3) increases in CD4+ and CD8+ T-cell counts among participants with the lowest baseline CD4+ T-cell counts. Together, these findings suggest that SBI warrants further exploration as a potential intervention to improve gut integrity and decrease inflammation.

SBI likely works by multiple mechanisms. SBI may bind luminal microbial antigens, preventing their translocation into the lamina propria via steric exclusion and preventing their ability to activate macrophages and dendritic cells [13, 14]. Indeed, SBI can bind bacterial, viral, and fungal microbe-associated molecular patterns. The ensuing lack of antigenic stimulation may explain why SBI has been shown to decrease leukocyte recruitment into the lamina propria [20]. Much like with intravenous immunoglobulin, binding of the Fc portion of IgG in SBI to Fc receptors on target T cells may enhance its anti-inflammatory effect [14]. SBI has also been shown to increase the abundance of *Proteobacteria Burkholderiales, Firmicutes Catonella*, and other bacteria in the small intestine [21] that may be associated with improvement in gut health [22]. A similar intervention, purified protein concentrate from plasma, has been shown to increase tight junction formation in weaning piglets and in a rat model of intestinal colitis [14]. Amino acids in SBI such as glutamine may further facilitate epithelial healing [14]. Whether growth factors are present in SBI that could stimulate T-cell proliferation, accounting for the increase in CD4+ T cells, warrants future determination. Thus, use of bioactive proteins as found in SBI may improve intestinal epithelial barrier function through immunomodulation, alteration of gut bacteria, and increasing tight junction formation [23].

Zonulin opens tight junctions reversibly in response to small intestinal exposure to enteric bacteria such as *E. coli* [24]. The small intestine has a low bacterial burden (up to 10^4^ colony forming units [CFU]/μL) in healthy people, predominantly Gram-positive aerobic bacteria proximally and facultative anaerobes distally, whereas the colon contains 10^12^ CFU/μL of strict anaerobes [25]. Small intestinal colonization by colonic flora upregulates zonulin and consequently increases intestinal permeability [25]. Lower zonulin levels have been associated with more bacterial diversity, suggesting the composition of the small intestinal bacteria impacts zonulin levels [26]. Zonulin levels are higher in Crohn's disease [27] and experimental cholera [28], and other intestinal diseases, indicating that in most circumstances, high zonulin levels reflect intestinal disease. Unexpectedly, in a previous study of people with HIV, higher zonulin levels were associated with lower mortality risk in a population with low current and nadir CD4+ T-cell counts (median nadir 30 cells/mm^3^) [2]. The high zonulin levels in survivors in this study may reflect the preservation of cells capable of producing zonulin and therefore better gut health rather than increased tight junction damage. In contrast, our population had higher CD4+ T-cell counts and normal nutrient absorption; therefore, the decrease in zonulin levels we observed may reflect a decrease in stimulation, such as dysbiosis and bacterial overgrowth, rather than a decrease in the cells capable of producing the protein. The decrease in I-FABP levels, which has been associated with decreased mortality,[2] reflects decreased enterocyte turnover, consistent with our hypothesis and previous findings [15]. Levels of I-FABP increase from acute to chronic HIV infection and, in contrast to many biomarkers predictive of poor outcomes, increase further with ART [29]. The simultaneous decrease in I-FABP and zonulin suggests that SBI decreases enterocyte turnover. Whether this reflects an improvement in gut health is unclear, but the simultaneous decrease in IL-6 indicates it may be beneficial.

Contrary to our hypothesis, no direct marker of microbial translocation, including LPS, flagellin, and 16S rDNA, was affected by SBI treatment. Plasma markers of microbial translocation decrease with long-term ART [3], so it is possible that our study was not sufficiently powered to detect further reductions in these markers. As improved gut health would need to precede changes in microbial translocation, this study may have been too short to detect decreases in these circulating markers. Moreover, several factors are known to interfere with LPS measurement [1], leading to the suggestion that indicators of the host response to LPS may be more accurate and relevant than measurement of LPS itself. The lack of effect on sCD14 levels, one such indicator, suggests that prevention of microbial translocation may not be the primary mechanism by which SBI can decrease inflammation.

The decrease in IL-6 levels observed in HIV participants following 24 weeks of oral SBI, accompanied by decreases in I-FABP and zonulin but not microbial translocation markers, suggests that 1) the upstream mediator of gut damage also upregulates IL-6 production, 2) increased IL-6 production may disrupt the gut barrier, or 3) increased gut barrier dysfunction stimulates IL-6 production. Whether these findings are due to alterations in the gut microbiome that facilitate epithelial healing and decrease inflammation, immunomodulatory proteins in SBI, or decreased translocation of microbial products not detected in the plasma is unknown. Some studies have suggested immune activation and inflammation improve during clinical studies due to increased participant adherence [30-32]. Excellent self-reported adherence including at entry and persistently undetectable HIV-1 RNA levels suggest that improved adherence is unlikely to contribute to the decreased inflammation observed here, although we did not measure HIV-1 RNA by single-copy assay. Assuming that the decrease in IL-6 is due to SBI and given that higher IL-6 levels are associated with increased atherosclerosis and cardiovascular disease [33, 34], AIDS events [35], non-AIDS events [36], and mortality [2, 34, 37], an intervention that decreases its levels could have a profound impact on clinical outcomes in HIV infection. We demonstrated a 38% decrease in IL-6 levels in participants in the first quartile; IL-6 levels were about 40% higher in participants who died compared to controls in the SMART study [38] and about 40% higher in immune non-responders compared to immune responders [39], suggesting the difference observed here may be clinically relevant. Further investigations into the mechanism of action may highlight ways to improve SBI's efficacy and potentially ways to synergize with other interventions.

The increase in CD4+ T cells in participants with the lowest quartile of CD4+ T cells at baseline is unlikely to reflect increased adherence as noted above [31]. One possibility is that this increase is due to decreased immune activation by SBI. Immune activation has been associated with poor CD4+ T-cell recovery [2], and immunosuppression with prednisolone in the absence of ART can improve CD4+ T-cell counts [40]. SBI may decrease apoptosis of activated CD4+ T cells [41] or downregulate adhesion molecules on the surface of CD4+ T cells [41], allowing egress of CD4+ T cells from tissues into the circulation [42]. Alternatively, immunoglobulins or other factors in SBI may increase de novo thymic output of CD4+ cells or expansion of CD4+ T-cell populations [43]. The parallel findings in CD8+ T cells suggest that the mechanism is not CD4+ T-cell specific, and it is not likely to be mediated by changes in residual HIV replication. These findings, however, could reflect regression to the mean, and the changes observed may not be due to any effect of SBI. Evaluating SBI in a longer, randomized, placebo-controlled trial in immunologic non-responders is essential for determining whether it has any effect on CD4+ T-cell recovery.

Other agents have been tested to improve gut integrity and decrease microbial translocation and inflammation in chronic HIV infection. Most agents targeting gut health had little if any effect, including sevelamer carbonate [44], rifaximin [45], mesalamine [46], and prebiotics and probiotics [47]. Immunomodulatory agents such as atorvastatin [48] have also been disappointing. Notably, hydroxychloroquine decreased IL-6 and LPS levels in immunologic non-responders [49], corticosteroids decreased IL-6 in viremic participants receiving ART [50] and sCD14 in untreated participants [40], and rosuvastatin, in contrast to atorvastatin, decreased sCD14 levels [51]. Recently, canakinumab, an IL-1b antibody, was shown to decrease IL-6 levels in a pilot study of people receiving suppressive ART [52]. However, these drugs operate systemically with pleio-tropic effects and are not without the potential for serious adverse events. Gut-targeted treatment with oral SBI might be a safer means to decrease inflammation.

This study had several limitations. First, there was no placebo arm for the entire duration of the study. Thus, it is possible that the changes observed at 24 weeks could be due to continued ART alone, although previous literature suggests stability of these biomarkers given the duration of ART [3, 45, 46], and this population had a median duration of ART of >8 years. Second, the increases in peripheral CD4+ T-cell counts were observed during post hoc analysis and may reflect regression to the mean. Therefore, these results need to be interpreted cautiously. Third, study participants had a wide range of duration of virologic suppression (1-23 years), which could have influenced the changes observed [41]. Fourth, participants spanned a wide age range (32 to 70 years); older individuals have more immune activation [53] and are at higher risk for persistently reduced CD4+ T-cell counts during ART [54]. Lastly, numerous correlations were assessed and no adjustments were performed, which has been supported for exploratory analyses [17], but it is possible the positive findings could reflect noise rather than true biological associations.

In conclusion, our results demonstrate that oral administration of SBI improved markers of gut barrier function and systemic inflammation. A longer, randomized, placebo-controlled trial of SBI in people with HIV and immunologic failure is needed to assess the clinical impact and mechanisms of action of SBI.

## SUPPLEMENTARY MATERIAL

**Supplementary Table 1.** Within-Group Comparisons for Randomized Treatment Groups^a^ at Week 4

Variable	Treatment Group	n Median (Q1-Q3)						P-value^b^
		Baseline		Week 4		Delta		
CD4 (cells/uL)	Placebo	35	523 (397–921)	35	635 (330–858)	34	-12.50 (-133–58)	*0.396*
	SBI 2.5 g	34	813 (469–939)	33	730 (483–1073)	33	0 (-96–119)	*0.752*
	SBI 5.0 g	33	672 (478–776)	31	597 (423–723)	31	-42 (-115–58)	*0.109*
								
CD4/CD8	Placebo	35	0.69 (0.46–1.22)	35	0.76 (0.50–1.17)	34	0 (-0.06–0.07)	*0.667*
	SBI 2.5 g	34	0.76 (0.42–1.22)	33	0.80 (0.47–1.29)	33	0 (-0.05–0.04)	*0.992*
	SBI 5.0 g	33	0.68 (0.53–1)	31	0.73 (0.48–0.89)	31	0 (-0.06–0.07)	*0.880*
								
I-FABP (ng/mL)	Placebo	35	1.94 (1.10–3.32)	35	1.74 (1.12–3.13)	34	0 (-0.76–0.68)	*0.910*
	SBI 2.5 g	34	1.69 (0.99–2.69)	31	1.54 (1.03–2.12)	31	-0.11 (-0.75–0.55)	*0.592*
	SBI 5.0 g	33	1.81 (1.12–2.66)	31	2.07 (1.19–2.68)	31	0.30 (-1.08–1.07)	*0.674*
								
Zonulin (ng/mL)	Placebo	35	34.76 (28.39–44.63)	35	36.28 (24.48–51.49)	34	-3.06 (-11.14–10.91)	*0.913*
	SBI 2.5 g	34	33.65 (18.89–41.77)	31	34.84 (25.23–46.11)	31	3.28 (-13.42–14.13)	*0.954*
	SBI 5.0 g	33	36.07 (22.87–49.35)	31	26.54 (20.37–45.94)	31	-5.90 (-14.69–9.21)	*0.335*
								
Flagellin (ng/mL)	Placebo	35	5.01 (3.87–8.63)	33	4.79 (2.28–7.95)	32	-0.09 (-2.42–2.49)	*>0.999*
	SBI 2.5 g	34	4.06 (2.21–5.98)	31	5.15 (2.62–7.20)	31	0 (-1.42–2.74)	*0.533*
	SBI 5.0 g	33	4.93 (2.97–7.42)	29	4.91 (2.56–7.24)	29	-0.11 (-2.37–0.76)	*0.360*
								
sCD14 (ng/mL)	Placebo	35	1.84 (1.59–2.04)	35	1.88 (1.52–2.24)	34	-0.03 (-0.19–0.22)	*0.848*
	SBI 2.5 g	34	2.12 (1.70–2.28)	31	1.95 (1.65–2.30)	31	-0.13 (-0.38–0.26)	*0.340*
	SBI 5.0 g	33	1.89 (1.48–2.23)	31	1.81 (1.49–2.22)	31	0.02 (-0.22–0.26)	*0.554*
								
IL-6 (pg/mL)	Placebo	35	1.29 (0.91–2.82)	35	1.25 (0.86–1.83)	34	-0.23 (-1.69–0.47)	*0.045*
	SBI 2.5 g	34	1.79 (1.31–2.71)	31	1.37 (0.82–1.83)	31	-0.46 (-1.09–0.49)	*0.194*
	SBI 5.0 g	33	1.45 (0.91–2.36)	31	1.20 (0.32–2.30)	31	-0.25 (-0.53–0.70)	*0.776*

a Analysis population = Participants randomized to LD-SBI, HD-SBI or PBO at initiation of study

b Wilcoxon Signed-Rank test

Abbreviations: SBI, Serum-derived bovine immunoglobulin/protein isolate; I-FABP, intestinal fatty acid binding protein; sCD14, soluble CD14
